# Digital Ageism: Challenges and Opportunities in Artificial Intelligence for Older Adults

**DOI:** 10.1093/geront/gnab167

**Published:** 2022-01-20

**Authors:** Charlene H. Chu, Rune Nyrup, Kathleen Leslie, Jiamin Shi, Andria Bianchi, Alexandra Lyn, Molly McNicholl, Shehroz Khan, Samira Rahimi, Amanda Grenier

**Affiliations:** 1 Lawrence S. Bloomberg Faculty of Nursing, University of Toronto, Toronto, Ontario, Canada; 2 KITE—Toronto Rehabilitation Institute, University Health Network, Toronto, Ontario, Canada; 3 Leverhulme Centre for the Future of Intelligence, University of Cambridge, Cambridge, UK; 4 Faculty of Health Disciplines, Athabasca University, Athabasca, Alberta, Canada; 5 Dalla Lana School of Public Health, University of Toronto, Toronto, Ontario, Canada; 6 University Health Network, Toronto, Ontario, Canada; 7 University of Cambridge, Cambridge, UK; 8 London School of Hygiene and Tropical Medicine, University of London, London, UK; 9 Institute of Biomedical Engineering, University of Toronto, Toronto, Ontario, Canada; 10 Department of Family Medicine, McGill University, Montreal, Quebec, Canada; 11 Mila—Quebec AI Institute, Montreal, Quebec, Canada; 12 Factor-Inwentash Faculty of Social Work, University of Toronto, Toronto, Ontario, Canada; 13 Baycrest Hospital, Toronto, Ontario, Canada

**Keywords:** Bias, Gerontology, Machine learning, Technology

## Abstract

Artificial intelligence (AI) and machine learning are changing our world through their impact on sectors including health care, education, employment, finance, and law. AI systems are developed using data that reflect the implicit and explicit biases of society, and there are significant concerns about how the predictive models in AI systems amplify inequity, privilege, and power in society. The widespread applications of AI have led to mainstream discourse about how AI systems are perpetuating racism, sexism, and classism; yet, concerns about ageism have been largely absent in the AI bias literature. Given the globally aging population and proliferation of AI, there is a need to critically examine the presence of age-related bias in AI systems. This forum article discusses ageism in AI systems and introduces a conceptual model that outlines intersecting pathways of technology development that can produce and reinforce digital ageism in AI systems. We also describe the broader ethical and legal implications and considerations for future directions in digital ageism research to advance knowledge in the field and deepen our understanding of how ageism in AI is fostered by broader cycles of injustice.

The intersection of an aging population with rapid technological advancements has given rise to novel considerations in the realm of Artificial Intelligence (AI). As defined by Russel and Norvig, AI is the "study of agents that receive percepts from the environment and perform actions" (Russell & Norvig, 2010, p. viii).

Current research examining biases in AI is largely focused on racial and gender biases and the serious consequences that arise as a result (Zhavoronkov et al., 2019); however, little attention has been paid to age-related bias (known as ageism) in AI (Butler, 1969). Ageism is a societal bias conceptualized as (a) prejudicial attitudes toward older adult populations and the process of aging, (b) discriminatory practices against older adults, and/or (c) institutionalized policies and social practices that foster these attitudes and actions (Rosales & Fernandez-Ardevol, 2020; Wilkinson & Ferraro, 2002). The pervasiveness of ageism has been highlighted in the coronavirus disease 2019 (COVID-19) pandemic where older adults were considered to be the most sick and vulnerable population (Vervaecke & Meisner, 2021). A report from the World Health Organization (WHO) and United Nations (UN) calls for urgent action to combat ageism due to its negative impacts on well-being, premature death, and higher health costs (WHO & UN, 2021). As noted in the WHO and UN (2021) report, scarce health care resources are sometimes allocated based on age, which means that an individual’s age may influence whether or not they receive an essential health intervention(s). With biases in AI recognized as a critical problem requiring urgent action, it is essential to invest in evidence-based strategies to prevent and tackle age-related bias in AI systems. These strategies can inform future legal and social policy developments to help mitigate this bias and advance social equity. In this Forum, we introduce the term *digital ageism* that we define as age bias in technology such as AI and discuss the mechanisms that lead to biases in AI systems. In the subsequent sections, we describe ageism in AI systems, broader ethical and legal implications, and considerations for future directions in research.

## Biases in AI Systems

AI has experienced exceptional advancements in its ability to learn and reason and accordingly has been described as the "fastest-moving technology" (Brown, 2020). As a tool, there are no inherent limits to the potential range of uses for AI. At their most fundamental, AI tools work by subjecting large data sets—the bigger the better—to rapid machine learning algorithms capable of pattern recognition, statistical correlation, prediction, inference, and problem-solving (Presser et al., 2021). A recent report indicates that a "digital world" of more than 2.5 quintillion bytes of data is produced each day (O’Keefe et al., 2020). As a result of its immense capability to process data for predictive modeling, AI has been touted for its transformative potential and has become increasingly salient as a matter of public and political interest. The ability of AI to supplement human decision making at super speed and on a large population or global scale positions AI to fundamentally change the nature of the global economy (Margetts & Dorobantu, 2019; Presser et al., 2021).

Notwithstanding its immense promise, AI applications released to the public are not free from racial and gender biases (Chen, Szolovits & Ghassemi, 2019; Howard & Borenstein, 2018). For instance, a widely deployed AI algorithm was shown to underestimate the health risks of Black patients compared to White patients (Obermeyer et al., 2019). The algorithm’s prediction was based on individuals’ health care costs, but it failed to consider the primary cause of Black patients’ lower spending on health care which is reduced health care access due to systemic racism. Other instances of racial bias include AI systems assigning longer jail sentences to Black inmates (Angwin et al., 2016) and imprecise facial recognition algorithms misidentifying Black faces at a 5 times higher rate than White faces (Simonite, 2019). AI bias against women has also been identified with serious socioeconomic consequences including women being less likely to receive job search advertisements for high-paying positions (Dastin, 2018) and job discrimination (Datta et al., 2015). This bias can be attributed to the way AI’s predictive algorithms learn from not only quantitative data but also text (i.e., corpus), which insidiously encodes historical–cultural associations that result in semantic biases, such as associations between stereotypical male names and working in the labor force or, conversely, female names and family/child-rearing (Caliskan et al., 2017).

**Figure 1:**
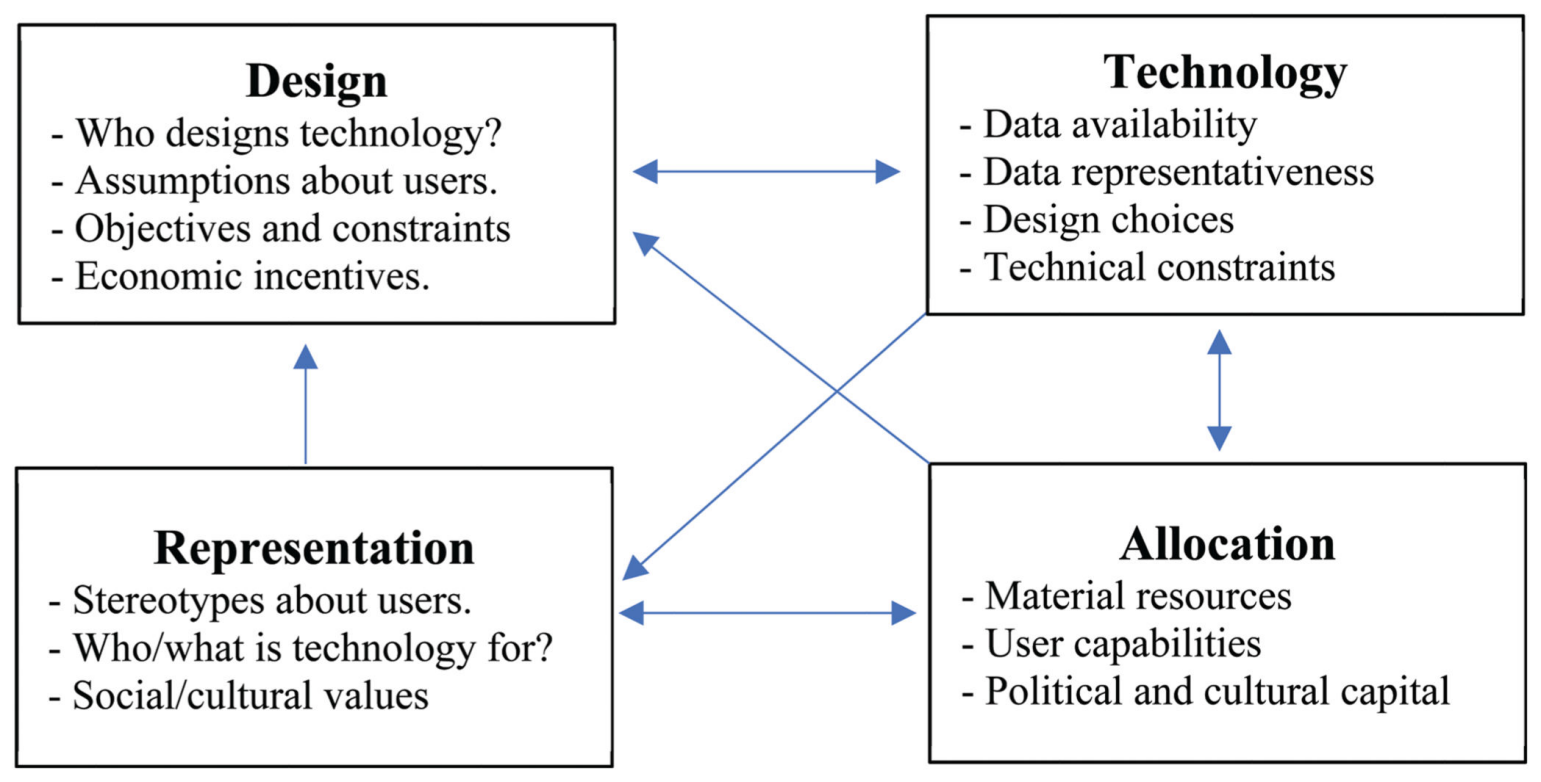
Cycles of injustices in how technology is developed, applied, and understood by members of society (Whittlestone et al., 2019).

One of the earliest definitions of bias in computer systems refers to a system’s ability to "systematically and unfairly discriminate against certain individuals or groups of individuals in favor of others. A system discriminates unfairly if it denies an opportunity or a good or if it assigns an undesirable outcome to an individual or group of individuals on grounds that are unreasonable or inappropriate" (Friedman & Nissenbaum, 1996, p. 332). Two unique types of undesirable outcomes can result from algorithmic bias: harms of allocation and harms of representation (Crawford, 2017). *Harms of allocation* refer to the distribution of resources and opportunities. This includes opportunities like when to be released on bail, receiving notification about potential job prospects, and access to health care resources or services. In contrast, *harms of representation* refer to how different groups or identities are represented and perceived by society. It is important to note that the underlying causes of these types of harms are complex. While technical factors, such as biased data and design choices, play an important role, biases can also arise from the context of use, for example, how human users interpret system outputs or from a mismatch between the capabilities and values assumed in the design of the system and those of its actual users (Danks & London, 2017; Friedman & Nissenbaum, 1996). These contextual factors can reflect underlying individual and social biases from as early on as technology inception, like who is involved in the design of technologies and the assumptions they make about end-users, to technology use by end-users, who have discrepancies in resources and capabilities to use existing technologies that affect what kind of data (and about whom) is readily collected. All of these factors are in turn shaped by both the allocative and representational effects of existing technologies, potentially creating a "cycle of injustice" (Whittlestone et al., 2019), where technological, individual, and social biases interact to produce and mutually reinforce each other (Figure 1). In the literature examining biases in AI, age-related bias is seldom discussed in comparison to racial and gender biases. It is time to critically reflect on and consider the experience of ageism in AI: the process of growing old in an increasingly digital world that directly and insidiously reinforces social inequities, exclusion, and marginalization. The next sections will focus on the digital divide, cycles of injustice that reinforce ageism, and the ethical and legal aspects of digital ageism.

## Ageism and the Digital Divide

Both the development and use of technology have excluded older adults, producing a "physical-digital divide," which exists when a group feels ostracized when they are unable to engage with the technologies being used around them (Ball et al., 2017). The social exclusion of older adults from the development and use of digital platforms results in data symptomatic of age-related bias in AI (Rosales & Fernandez-Ardevol, 2020; Wilkinson & Ferraro, 2002). There is a misconception that older adults are a homogenous group of people who are "in decline," incompetent, and in need of younger people’s guidance when it comes to technology (Mannheim et al., 2019). Furthermore, these paternalistic stereotypes and patronizing sentiments contribute to harmful *compassionate ageism*—"stereotypes concerning older persons that have permeated public rhetoric" (Binstock, 1983)—which is then reinforced and internalized by older adults (Vervaecke & Meisner, 2021). Internalized negative stereotypes can cause older adults to experience a decline in cognitive (e.g., memory) and psychological performances (Hehman & Bugental, 2015; Hess et al., 2003).

Furthermore, in a society where AI is becoming increasingly prevalent, older adults are at risk of further social exclusion and retrogression due to a digital divide (Rosales & Fernandez-Ardevol, 2020). The risk of a gap or distinction that delineates this aging population according to those with access to information technology and those without grows as technology advances (Srinuan & Bohlin, 2011). While older adults are using technology in greater numbers (Anderson et al., 2017) and benefitting from technology use (Anguera et al., 2017; Cotten et al., 2011; Czaja et al., 2018; Decker et al., 2019; Harerimana et al., 2019; Hurling et al., 2007; Irvine et al., 2013; Tomasino et al., 2017; White et al., 2002), they continue to be the least likely age cohort to have access to a computer and the internet due to physical barriers (e.g., physical disability) and/or psychological factors (e.g., lack of confidence to technology use; Anderson et al., 2017; Tomasino et al., 2017). One report from the European Union indicates that one third of older adults report never using the internet (Anderson et al., 2017). A survey of 17 European countries showed that internet use in older adults varied depending on location and age with the rates of internet nonusers increasing with each decade of age (König et al., 2018). Results show that 52% of individuals 65 years and older were internet nonusers and the percentage of internet nonusers increased to 92% in those 80–84 years old, indicating that "many older Europeans do not use the Internet and are particularly affected by the digital divide" (König et al., 2018, p. 626). Similarly, in Toronto, Canada, residents aged 60 and older report having lower rates of access to home internet compared to younger residents, with those who have access experiencing internet speeds below the Canadian national target of 50 Mbps (Andrey et al., 2021). Additionally, almost one third (30%) of this older adult cohort lack a device through which they can connect to the internet (Andrey et al., 2021). Older people may also experience more disparities in material access to technologies, education, and support to learn new technology (Ball et al., 2017; Cronin, 2003; Lagacé et al., 2015). For some older adults, the challenge to learn to use technology and the fear that technology will fail to work when most needed can be stressful (Cotten et al., 2011).

## Ageist Cycles of Injustice in Digital Technologies

The barriers to technological access outlined above provide insight as to possible explanations for the exclusion of older adults from the research, design, and development process of digital technologies (Baum et al., 2014; Kanstrup & Bygholm, 2019; Lagacé et al., 2015). Older adults are sometimes referred to as "invisible users" in the literature alluding to their exclusion in the process of technology design that makes their interests and values invisible (Kanstrup & Bygholm, 2019; Rosales & Fernandez-Ardevol, 2019). Their perspectives are unlikely or inaccurately taken into consideration during technology design or product development which are activities dominated by younger people. Research by Charness (1990, 1992, 2009, 2020) highlights a misalignment of person-system fit that is generated when normative age-related changes, like in perception, cognition, and psychomotor abilities, are not accounted for which contributes to older adults’ low adoption rates and sub-optimal user experiences. The impact of this mismatch will be intensified over time as society transitions to an increased use of technology (e.g., health care technologies, information and communication technologies) which leaves older adults further behind from a technology-enabled world.

Additionally, ageist attitudes (Abbey & Hyde, 2009), which manifest in marketing and research studies (Ayalon & Clemens, 2018), influence the design of technology through a historical exclusion of older adults, particularly at arbitrary upper age limits (50+ or 60+) (Mannheim et al., 2019). The perception of older adults as a homogenous group potentially results in a loss of recognizing the nuanced needs of older people. Moreover, a disproportionate amount of information technology targets older adults specifically for health care and chronic disease management (Mannheim et al., 2019), rather than for leisure, joy, or fun. The underlying assumption of this phenomenon is that older adults are unhealthy and that managing health conditions is the only reason that they may seek to use and benefit from technology. This assumption could consequently create a feedback loop that reinforces negative stereotypes. Specifically, if most technologies marketed toward older adults are designed to resolve or manage health problems, then this could easily reinforce the impression that older adults are mainly unhealthy, in need of support, and/or in decline. There is evidence of significant age bias as demonstrated by Díaz et al. (2018) who used sentiment analysis on a large corpus of text data from Wikipedia, Twitter, and web crawling the internet. Díaz et al. (2018) found age-related bias with respect to explicit and implicit encoded ageist stereotypes. For example, sentences containing "young" were 66% more likely to be scored positively than the same sentences containing "old" when controlling for other sentential content, and in their analysis of word embedding to explore implicit bias, they found "youth" was associated with words like "courageous" and the words "old" and "older" were associated with "stubborn" and "obstinate." Another effect is that the data collected from these technologies end up representing only a segment of older adults with health issues. This selection bias does not enable technologies to capture the heterogeneity of the aging population, causing a mismatch between targeted technology such as AI and the actual needs of older adults (Crawford, 2017).

Taken together, there is not enough data from older adults available for training AI models, and the corpus that is available shows an explicit and implicit age-related bias (Díaz et al., 2018). Problems arise when the corpus may be mined by algorithms to understand attitudes toward or about products or services, and the "sentiment output is less positive simply because the sentences describe an older person taking part in an interaction" (Díaz et al., 2018, p. 9). This can result in further bias that leads to nongeneralizable AI models and the development of future AI systems that ignore the use, interests, and values of older adults while reinforcing or amplifying existing disadvantages (Coiro, 2003). In addition, this bias could influence or reduce the products or services targeted for older individuals (Díaz et al., 2018).

**Figure 2:**
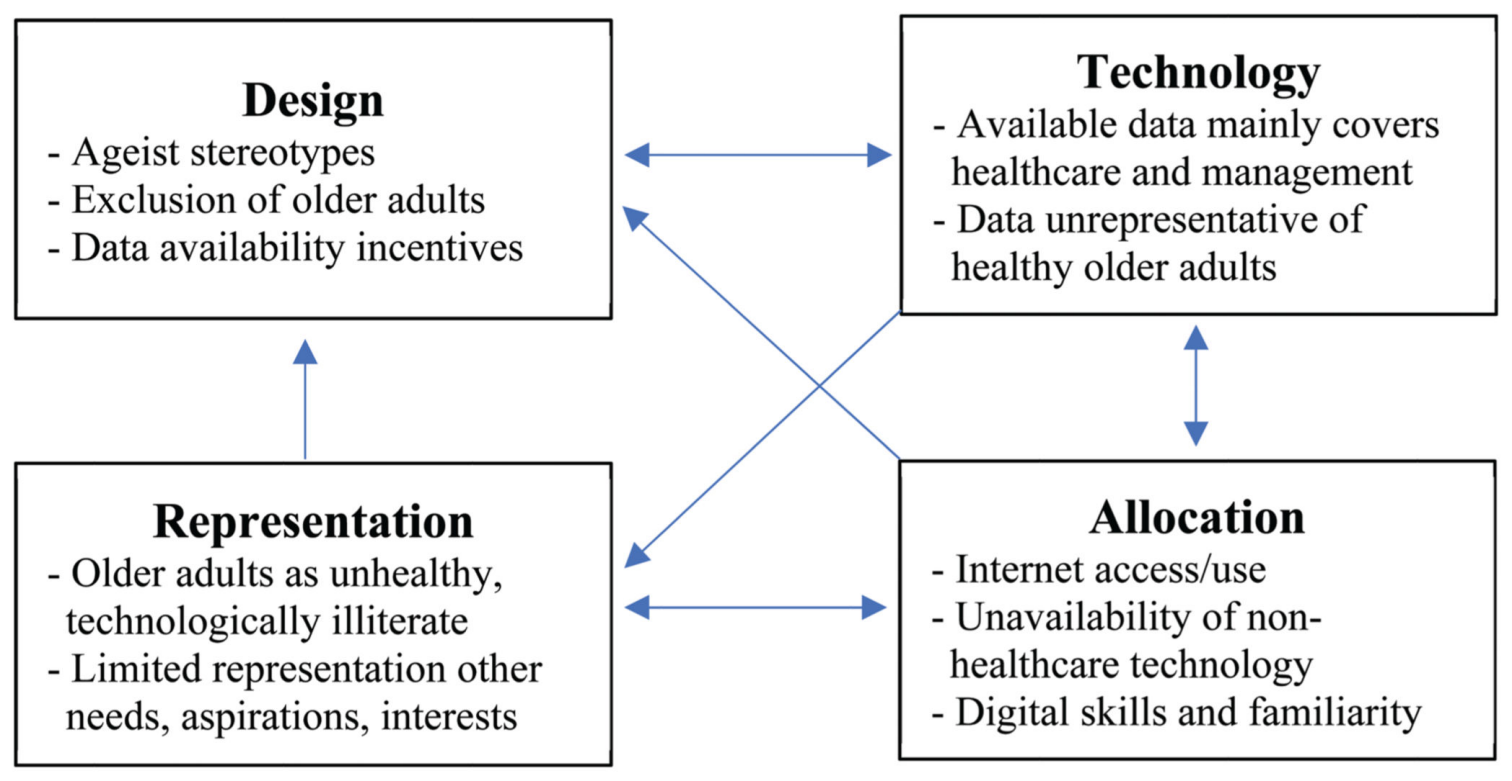
How cycles of injustice in digital technologies result in digital ageism.

AI systems can produce and reinforce ageist biases through multiple pathways. Addressing bias requires a deeper understanding of how ageism fits into a broader cycle of injustice as illustrated in Figure 2. Existing stereotypes of older adults as unhealthy and/or technologically incompetent (Representation) affect the assumptions made about older adults, which can lead to the exclusion of older adults from research and design processes (Design). Ageist stereotypes are further reinforced by the fact that new information technologies for older adults mostly focus on health and health care management (Design/Technology). The digital divide (Allocation), together with patterns in existing applications, results in data sets that inaccurately represent healthy older adults (Technology). These biased data sets incentivize further technology development that primarily focuses on health care needs (Design). The limited availability of digital technologies serving other needs, interests, and aspirations of older adults can further entrench the digital divide (Allocation).

In this way, new systems reinforce inequality and magnify societal exclusion for subsects of the population who are considered a "digital underclass" (Petersen & Bertelsen, 2017), primarily made up of older, poor, racialized, and marginalized groups. This raises questions about how older adults are included and viewed in our increasingly digital world, and how our societal structures that enforce ageism are represented in AI systems. There is a pressing need to address these foundational questions especially with the surge of digital technology use during the COVID-19 pandemic (De’ et al., 2020).

## Ethical and Legal Implications of Ageism in AI

Ageism is an overlooked bias within AI ethics. This is evident upon our search of the AI Ethics Guidelines Global Inventory (AlgorithmWatch, 2021), a repository that compiles documents about how AI systems can conduct ethical automated decision making. Most of these guidelines highlight fairness as a key governing ethical principle; fairness typically incorporates considerations of equity and justice. In the repository, there are 146 documents created by government, private, civil society, and international organizations, which are accessible and available in English. The research team searched these documents for the terms ageism and similar concepts like age bias, age, old/older, senior(s), and elderly. We found that only 34 (23.3%) of these documents mention ageism as a bias for a total of 53 unique mentions. Of these, 19 (54.7%) merely listed "age" as part of a general list of protected characteristics. For example, the UNI Global Union Top 10 Principles for Ethical AI (2018) states "In the design and maintenance of AI, it is vital that the system is controlled for negative or harmful human bias, and that any bias—be it gender, race, sexual orientation, *age,* etc.—is identified and is not propagated by the system" (p. 8). Only 12 (8.2%) of the examined documents provided slightly more context about bias against older adults, often no more than one or two sentences. For example, the Academy of Royal Medical College’s Artificial Intelligence in Healthcare report (2019) states "It might be argued that the level of regulation should be varied according to the risks—for example psychiatric patients, the young and the elderly [sic] might be at particular risk from any ‘bad advice’ from digitised systems" (p. 28).

Ultimately, our overview of these documents demonstrates that ageism directed toward older adults is insufficiently recognized as a specific and unique ethical implication of AI in current literature. To ensure that AI is developed in an ethically defensible manner, such that it promotes equity and rejects unjust bias, this implication ought to be explicitly recognized and addressed. As indicated in previous sections of this Forum article, failing to appropriately involve and accurately represent older people leads to a digital divide that may further contribute to further preventable inequities.

One significant concern about failing to respond to ageism in AI relates to the presence of ageism in AI-powered hiring systems. Consider for example an AI-powered resume-screening tool that excludes job candidates based on their date of graduation. In 2017, AI-driven hiring platforms including Jobr were under investigation for prohibiting applicants from selecting either graduation year or any first job before 1980 (Ajunwa, 2019). Similarly, an algorithm may prioritize young, male applicants to reflect the current employee composition of an organization in an attempt to emulate the employer’s past hiring behavior, and in doing so, perpetuate preexisting biases (Kuei & Mixon, 2020). From an ethical and legal perspective, providing people with a fair opportunity is often considered an important part of what it means to treat people equally and justly (UN, 1945). Failing to provide suitable individuals with the ability to pursue a career opportunity on the basis of immutable characteristics (e.g., graduation year, gender) with no bearing on ability directly opposes the fair equality of opportunity principle.

The widespread use of AI tools to make recommendations with transformative consequences for individuals and society has given rise to an "urgent set of legal questions and concerns" (Presser et al., 2021). These concerns include security, fairness, bias and discrimination, legal personhood, intellectual property, privacy and data protection, and liability for damages (Rodrigues, 2020). There is growing recognition of the need for "normative frameworks for the development and deployment of AI" (Martin-Bariteau & Scassa, 2021). Regulatory governance frameworks are important in preventing and mitigating harm occasioned by the deployment of AI algorithms and can outline the legal recourse available to an aggrieved individual or entity. In the development context, regulatory governance frameworks provide guidance for the ethical development and deployment of AI (including recognizing and minimizing embedded bias).

In recent years, a wave of lawsuits has plagued major employers like Google and LinkedIn who used software algorithms to target internet job advertisements to younger applicants, excluding applicants older than 40 years (Ajunwa, 2018). There have also been multiple lawsuits and settlements based on Facebook’s paid advertisement platform, which enabled advertisers to micro-target ads to exclude users based on protected classes, such as age, which are in violation of federal and state civil rights laws (American Civil Liberties Union, 2019). These discriminatory advertising practices prevented older people from seeing ads for job opportunities, ostensibly denying them the opportunity for employment.

Stakeholders and regulators face unique challenges in AI regulation and governance. There is no uniform global legal code for AI governance. International sources of AI law may be persuasive in other jurisdictions but will not be binding. This means that lawmakers may look internationally for guidance on how other states or countries have navigated the challenges posed by the proliferation of AI, but will ultimately have to develop and implement regulatory systems that accord with their own legal structures. For example, the proposed Canadian Digital Charter Implementation Act (2020) was modeled on the European Union’s General Data Protection Regulation (2016).

Developing laws and regulations regarding technology have global challenges and issues with regard to applications within and across country boundaries. For example, in the Canadian context, governments and regulators must grapple with regulating AI within our federal and constitutional setting (Martin-Bariteau & Scassa, 2021) because powers over health care and human rights are shared between federal and provincial governments. As a result, "[c] oherent, consistent and principled AI regulation in Canada [necessitates] considerable federal-provincial co-operation as well as strong inter agency collaboration—both that may be difficult to count on" (Martin-Bariteau & Scassa, 2021). Beyond jurisdictional issues, governments have sought to balance competing regulatory interests, including the need to protect the public and the need to exercise regulatory restraint as to not stifle innovation (Martin-Bariteau & Scassa, 2021). Adding to this challenge, some AI algorithms are proprietary and thus are afforded intellectual property protections. These intellectual property protections have precluded aggrieved individuals (including criminal defendants) from having access to and examining the AI algorithm (see *State v Loomis* 881 N.W.2d 749 (Wis. 2016) 754 (US)). AI algorithms behind many social, political, and legal applications of AI have used intellectual property protections to avoid legal and research scrutiny.

Transparency and careful examination for age-related bias (such as through research) is required given the complexity of AI systems, without a deeper investigation we are not able to assess from a legal standpoint whether these systems are perpetuating the ageism that is pervasive in society. Ultimately, the concern is that AI will simply, "reproduce existing hierarchies and vulnerabilities of social relations…" with regard to age and in a manner that avoids scrutiny through obscurity and lack of transparency (Martin-Bariteau & Scassa, 2021). Even with its widespread adoption, there is very little training, support, auditing, or oversight of AI-driven activities from a regulatory or legal perspective (Presser et al., 2021), and Canada’s current AI regulatory regime is lagging (Martin-Bariteau & Scassa, 2021). With the regulation of AI in Canada in its relative infancy, it remains unclear as to whether existing legal frameworks are sufficient to protect or offer any meaningful recourse to those who are victims of ageist bias occurring because of the use of AI.

## Looking Ahead

Although much of the discussion about AI and bias has focused on its potential to cause harm, we are optimistic that AI can be developed to mitigate human bias. In the area of employment, for example, new AI-based hiring platforms can help overcome human recruiter bias by detecting qualified candidates who may be overlooked in traditional hiring processes that use resumes and cover letters (Wiggers, 2021). More research developing technologies are also being conducted with older adults (Chu et al., 2021; Harrington et al., 2018), but there is a need for continued analysis of the process to address aspects of ageism (Mannheim et al., 2019). Additionally, mitigating biases in health care is an area of gaining more attention. In this context, the validation of the representativeness of the data set is suggested as the best approach to combat algorithmic bias (Ho et al., 2020). Looking ahead, we remain optimistic that the bias of *digital ageism* can be acknowledged and addressed through a multifaceted approach. First and foremost, from the lens of critical gerontology, it is crucial to include older adults throughout the pipeline when developing AI systems. This will require addressing structural issues such as access, time, training, and the means to participate in research and development, as well as existing funding constraints of research grants and technology development (Grenier et al., 2021). Next, an interdisciplinary approach that includes gerontologists, social scientists, philosophers, legal scholars, ethicists, clinicians, and technologists who could work collaboratively and lend their expertise to address *digital ageism* is warranted. An interdisciplinary and critical examination of age as a bias is necessary to capture the full picture for effective AI deployment, especially under the context of the COVID-19 pandemic, where, in some jurisdictions, age was the sole criterion for health care access and lifesaving treatments (WHO & UN, 2021).

There is an urgency and opportunity to better understand and address *digital ageism.* To date, the AI developed may be insufficient to meet the needs of older adults and may prove to be exclusionary and discriminatory. However, there is also an opportunity to develop programs and mechanisms that include older adults and to delineate what is fair and ethical with regard to AI. This is especially the case given the sociocultural shift where more and more people will, and are expected to, incorporate technology into their lives to remain connected to our technology-enabled world. Projections show that older adults are likely to make up the largest proportion of technology (e.g., health related, information and communication) consumers in the future as today’s tech-savvy adults grow older (Foskey, 2001; Kanstrup & Bygholm, 2019; Rosales & Fernandez-Ardevol, 2019). The COVID-19 pandemic was a significant accelerator of technology use and uptake for day-to-day needs (e.g., online groceries, shopping, health care) and social communication. Such ubiquitous use of technology (De’ et al., 2020) indicates that there is an increased number of people who are likely to be both excluded from these means of communication and affected by implicit biases in current AI systems. Together, these conditions underscore the need for more research on *digital ageism.*

For future directions, our research team will establish a multiphase research program to further explore the extent of ageism in AI and develop insights about the potential for age-related bias in AI applications that can perpetuate social inequity for older adults. We aim to expand on the described conceptual framework of how older adults experience ageism in and through AI to raise broad awareness of this bias and contribute to a more socially conscious approach to AI development. As the current younger generation may have grown up with widespread access to information and communication technologies like computers, social media, and the internet (referred to as "digital natives" [International Telecommunication Union, 2013; UN]), it is expected that these tech-savvy end-users will have greater expectations for fair and just AI applications as older adults in the future. To meet these future expectations, our interdisciplinary team aims to create data sets with more representations of older adults for fair algorithm development of AI technologies like facial recognition. Furthermore, we will develop partnerships with older adults organizations, governments, AI researchers and developers, and other stakeholders to shape legal and social policy with the aim to reduce technology-driven exclusion and inequities for older adults.

## Conclusions

Ageism is a bias that currently remains understudied in AI research. The exclusion of older adults from technology development maintains a broader cycle of injustice including societal ageist attitudes and exacerbates the digital divide. Thus, we urge future AI development and research to consider and include *digital ageism* as a concept in the research and policy agenda toward building fair and ethical AI.
